# Remote ischemic conditioning improves neuropsychiatric symptoms in COVID-19 patients: a randomized clinical trial

**DOI:** 10.3389/fpsyt.2025.1674886

**Published:** 2026-02-20

**Authors:** Yaxuan Wu, Mengfan Li, Haiying Li, Hui Li, Tengqun Shen, Yuanyuan Liu, Guangming Wang, Hairong Sun, Zhenguang Li, Jinbiao Zhang

**Affiliations:** 1 Department of Neurology, Xichang People’s Hospital, Xichang, Sichuan, China; 2 Department of Neurology, Weihai Municipal Hospital, Cheeloo College of Medicine, Shandong University, Weihai, Shandong, China; 3 Shandong Second Medical University, Weifang, Shandong, China; 4 Guangtai Engineering Laboratory, Weihai, Shandong, China; 5 Department of Resident Standardized Training Management, Weihai Municipal Hospital, Cheeloo College of Medicine, Shandong University, Weihai, Shandong, China; 6 Medical Administration Department, Weihai Municipal Hospital, Cheeloo College of Medicine, Shandong University, Weihai, Shandong, China; 7 Affiliated Hospital of Shandong Second Medical University, Shandong Second Medical University, Weifang, Shandong, China

**Keywords:** cognitive impairment, COVID-19, inflammation, insomnia, remote ischemic precondition

## Abstract

**Background:**

Coronavirus disease 2019 (COVID-19) may induce cognitive impairment, insomnia, anxiety, and depression through secondary inflammatory reaction. Remote ischemic conditioning (RIC), a promisingly noninvasive intervention, can modulate the inflammatory reaction in target organs and offer organ protection.

**Methods:**

This randomized, placebo-controlled, double-blinded trial recruited 65 patients with COVID-19-related insomnia to investigate whether RIC can improve neuropsychiatric symptoms in COVID-19 patients. Participants were evaluated for neuropsychiatric symptoms including cognition (Beijing version of the Montreal Cognitive Assessment (MoCA)), sleep (Pittsburgh Sleep Quality Index (PSQI), 14-item Fatigue Scale (FS-14) and Epworth Sleepiness Scale (ESS)), anxiety (Hamilton Anxiety Scale (HAMA)) and depression (Hamilton Depression Scale (HAMD-17)). Serum neuronal-derived exosomes (NDEs) complement proteins and mitochondria proteins were extracted and quantified.

**Results:**

MoCA score was significantly ameliorated in the RIC group and the PSQI, FS-14, ESS, HAMA and HAMD-17 scores were remarkably decreased in the RIC group at 3 months (*P* < 0.001 for MoCA, PSQI, FS-14, and ESS; *P* = 0.003 for HAMA; *P* = 0.005 for HAMD-17). The interaction effect of time-by group was also different (*P* < 0.001 for MoCA, PSQI, FS-14, and ESS; *P* = 0.003 for HAMA). Furthermore, the PSQI, FS-14, ESS, HAMA, and HAMD-17 scores were statistically decreased in the RIC group at three months after the end of RIC treatment (*P* < 0.001 for MoCA, PSQI, FS-14, and ESS; *P* = 0.03 for HAMA; *P* = 0.027 for HAMD-17).

**Conclusions:**

Our study suggested that RIC may be a potential adjuvant therapy for neuropsychiatric symptoms after COVID-19.

**Clinical trial registration:**

## Introduction

Severe acute respiratory syndrome coronavirus 2 (SARS-CoV2) infection can cause acute viral pneumonia, somnipathy (1), cognitive impairment (2), anxiety, depression, and other long-lasting symptoms. The World Health Organization defines long-term coronavirus disease (COVID) as the occurrence of persistent symptoms, such as fatigue and cognitive impairment, after three months of SARS-CoV2 infection, or the appearance of the above clinical manifestations as new symptoms lasting for more than 2 months (3). Multiple meta-analyses have demonstrated that the prevalence of insomnia, cognitive impairment, anxiety, and depression symptoms after SARS-CoV2 infection is estimated to be 12%-47% (1, 4–6), 14%-58% (7–9) and 45%-48% (10), respectively. Previous studies have shown that coronavirus disease 2019 (COVID-19) related insomnia significantly increase the anxiety symptom during the COVID-19 pandemic (11). Li et al. also demonstrating that insomnia was positively associated with anxiety and depression symptom and may independently predicted the occurrence of anxiety and depression symptoms during the first wave of the COVID-19 pandemic in China (12).

Emerging evidence has shown that inflammatory response is involved in long-term COVID, especially the complement system (13) and mitochondria (14). Meanwhile, C5b-9 disrupts the cell membrane and damages the endothelial cell, thus activating the complement cascade reaction and aggravating neuroinflammation. Furthermore, the mitochondrial Ca^2+^ uniporter (MCU) and human sodium/calcium exchanger 1 (SLC8A1) regulate mitochondria Ca^2+^. Overexpression of structural proteins in SARS-CoV-2 selectively affects the release of Ca^2+^ from the endoplasmic reticulum, thus disrupting the homeostasis of the mitochondrial Ca^2+^ signaling pathway, which increases the level of reactive oxygen species (ROS) and exacerbates neuronal apoptosis (15–17). Previous studies have shown that C5b-9 expression significantly increases in long-term COVID patients compared with patients who recover before 6-month follow-up (13).

Remote ischemic conditioning (RIC), a promising noninvasive prevention, can protect the endothelium against injury by modulating systematic inflammation and oxidative stress. It can also enhance cognitive abilities in individuals with vascular impairments and alleviate insomnia associated with Parkinson’s disease (18–20). Exosomes are small cell membrane-like structures with the potential to cross the blood-brain barrier (BBB), transporting information and materials between cells (21–23). Produced by neurons, astrocytes and microglia cells within the central nervous system (CNS), exosomes serve as valuable biomarkers reflecting the actual condition of the CNS (24, 25).

The current research on COVID-19 mainly focuses on the impact of SARS-CoV2 on the body, with only a few treatment-related studies. This study aimed to explore whether RIC can improve insomnia, cognitive impairment, anxiety, and depression in COVID-19 patients as well as the dynamic changes in C5b-9, complement factor B (CFB), MCU, and SLC8A1 in serum neural-derived exosomes (NDEs) in COVID-19 patients with insomnia before and after RIC treatment.

## Materials and methods

### Study design

This study was a prospective, single center, double-blinded, randomized clinical trial conducted in accordance with the Helsinki declaration. This study was approved by the ethical committee of Weihai Municipal Hospital (2023014). All participants provided written informed consent. A 1:1 randomized parallel-group design was used. The randomized clinical trial adhered to the Consolidated Standard of Reporting Trials (CONSORT) guideline and the trial protocol is detailed in **Supplementary File 1**.

### Study participants and sample size

This randomized clinical trial enrolled COVID-19 patients with insomnia treated at Weihai Municipal Hospital from May 2023 to May 2024. COVID-19 was diagnosed based on ORF1ab gene and N gene positive of the new coronavirus nucleic acid test. The inclusion criteria were: (1) Patients who developed insomnia three months after COVID-19 and met the diagnosis criteria of the International Classification of Sleep Disorders, Third Edition (26); (2) Patients aged 30–70 years with no history of neuropsychological assessment; (3) The participants who signed informed consent forms. Notably, the patients could not use other interventions to treat somnipathy after enrollment.

The exclusion criteria were: (1) Patients with chronic insomnia before COVID-19 according to the diagnostic criteria of the 2017 edition of the Chinese Adult Insomnia Diagnosis and Treatment Guidelines (27); (2) Patients with dementia before enrollment (the score of the Informant Questionnaire on Cognitive Decline in the Elderly ≥ 3.19) (28); (3) Patients with bleeding or major surgery, such as aortic or carotid before enrollment; (4) Individuals who experienced fever within the week prior to enrollment or had severe hypertension that was difficult to manage (defined as systolic blood pressure exceeding 180 mmHg or diastolic blood pressure above 110 mmHg despite medication) were excluded; (5) Patients with distal ischemic adaptation contraindications, such as significant soft tissue damage, fractures, or vascular injuries in the upper limbs, peripheral vascular disorders in the distal regions, or platelet counts below 100 × 10^9^/L.; (6) Patients with severe heart or blood system diseases, thyroid disease, malignant tumor or immune system disease or pregnant; (7) Patients whose laboratory test indicators did not qualify (aspartate aminotransferase or alanine aminotransferase higher than the upper limit of normal (thrice); creatinine clearance < 0.6 ml/s; serum creatinine > 265 umol/L (> 3.0 mg/dl); (8) Patients participating in other clinical studies, or had participated in other clinical researchers within 3 months before enrollment, or showed poor treatment compliance; (9) Patients taking any of the following drugs that may affect the nervous system: antidepressants, anxiolytics, antipsychotics, glucocorticoids or partial bronchodilators, antiepileptic and may influence cognitive function or sleep drugs (such as benzodiazepines, non-benzodiazepines, melatonin or melatonin receptor agonists, muscular relaxant, opioid analgesics, barbital, antihistamines, acetylcholinesterase inhibitor, dopamine agonist, monoamine oxidase inhibitor, NMDA receptor antagonists, levodopa, citicoline, piracetam, benzhexol).

A superiority test was performed with 80% power and a 5% significance level (α = 0.05), assuming a standard deviation of 10 for the neuropsychological score difference between the RIC and sham RIC groups. To address potential dropout rates, 73 participants were recruited, with 36 allocated to each group. The sample size was determined using G*Power 3.1 software.

### Randomization and blinding

Patients were randomly assigned to either the RIC treatment group or the sham RIC treatment group using a simple randomization method without stratification. A professional statistician at Weihai Municipal Hospital generated the randomization sequence using SPSS software, with the study sequence concealed in an airtight envelope. Participants and random sequence generator, as well as allocator and evaluators are independent of each other and are not aware of the grouping distribution.

### Clinical data

An experienced physician gathered demographic information using a standardized questionnaire. Hypertension and diabetes mellitus were diagnosed based on the National Institute for Health and Care Excellence guidelines (29) and the American Diabetes Association criteria (30), respectively. Hyperlipidemia was defined as either a prior diagnosis of hyperlipidemia or meeting the following criteria: low-density lipoprotein cholesterol ≥ 130 mg/dl, triglycerides ≥ 150 mg/dl, and total cholesterol ≥ 200 mg/dl.

### Magnetic resonance data acquisition

all participants underwent magnetic resonance imaging with a 3T scanner (Siemens, Erlangen, Germany). The examination sequence included T1-weighted and T2-weighted fluid attenuated inversion recovery (FLAIR) images, apparent diffusion coefficient (ADC) images and diffusion-weighted imaging (DWI) images (repeat time (TR) 4040 ms, echo time (TE) 64 ms, field of view (FOV) 220 × 220, matrix size = 512 × 512, section thickness = 5 mm). SWI images were acquired with TR = 29 ms, TE = 20 ms, flip angle = 15°, matrix = 448 × 168, FOV = 230 × 173 mm, and slice thickness = 2.0 mm. All the above sequence scans were completed by a professional radiologist.

Patients exhibiting any of the following conditions were excluded from this study: (1) patients presenting with hemorrhagic or ischemic stroke or transient ischemic stroke; (2) patients presenting with traumatic brain injury or brain tumors; (3) patients presenting with neurodegenerative disease (such as Alzheimer’s disease, Parkinson’s disease, multiple system atrophy, dementia with Lewy bodies, hydrocephalus, small vessel disease or vascular dementia; (4) patients who cannot complement the magnetic resonance imaging examination.

### RIC treatment procedure

RIC equipment from Weihai Guangtai Medical Technology Co. was used in this study. The operation was conducted as follows: bilateral upper arm cuff inflation for 5 minutes, then deflation for 5 minutes. (repeated five times for 50 minutes). The inflation pressure of the upper arm cuff in the RIC treatment group was 200 mmHg, and they continuously received treatment for three months (180 sessions, 2 sessions/day). Similarly, the inflation pressure of the upper arm cuff in the sham-treatment group was 60 mmHg, and they received treatment for 3 months (180 sessions, 2 sessions/day).

### Neuropsychiatric assessment (neuropsychological and sleep)

All participants were assessed face-to face by an experienced neurologist using a standard sleep and psychiatry scale, the cognitive impairment and depression and anxiety was diagnosis by a medical staff. Cognitive function was assessed using the Beijing version of the Montreal Cognitive Assessment (MoCA). The assessment includes cognitive items, such as visuo-executive functions, naming, attention, language, abstraction, delayed recall, and orientation. The cutoff score between cognitive impairment and non-cognitive impairment was 25, according to the revised criteria (31). Depression was assessed using the Hamilton Depression Scale (HAMD-17) (diagnosis criteria score on the HAMD-17 ≥ 17). Anxiety was assessed using the Hamilton Anxiety Scale (HAMA) (diagnosis criteria score on the HAMA ≥ 14). The sleep disorders were diagnosis by a medical staff following the diagnosis criteria of the International Classification of Sleep Disorders, Third Edition, and using the Pittsburgh sleep quality index (PSQI) and the 14-item Fatigue Scale (FS-14) (total score: 14) and the Epworth Sleepiness Scale (ESS) (total scores ≥ 10) to quantify. Sleep patterns, including sleep quality, sleep latency, sleep duration, sleep efficiency and insomnia, sleep medication, and daytime dysfunction, were assessed using PSQI. A PSQI score greater than 5 indicated poor quality of sleep. Fatigue, including physical fatigue (items 1-8) and mental fatigue (items 9-14), was assessed using FS-14. Higher scores indicated severe fatigue. The comprehensive daytime sleepiness level was assessed using ESS. Higher scores indicated excessive daytime sleepiness (EDS). Neuropsychological and sleep assessments were performed before RIC treatment, at 1 and 3 months after RIC or sham RIC treatment. Besides, the above assessments were conducted after 6 months of enrollment (the third month after the end of RIC treatment or sham RIC treatment). The assessments were conducted by a professional examiner blinded to the clinical data.

### Extraction of NDEs and quantification of C5b-9, CFB, MCU and SLC8A1

Fasting peripheral blood samples were drawn in the morning by a skilled technician using consistent methods and procedures. The samples were centrifuged at 4000 × g for 10 minutes within 30 minutes of collection. The resulting serum was separated and promptly stored at −80°C for subsequent analysis.

Specific NDEs were isolated as described in the published protocol (32). Using Enzyme-linked immunosorbent assay (ELISA) to quantify NDE proteins, Tetraspanning exosome marker CD63 (RayBio, ELH-CD63, Norcross, GA 30092, USA), complement fragment C5b-9 (Abbexa Ltd, abx054346, Cambridge, UK) and decay acceleration CFB (Abbxa Ltd, abx055457, Cambridge, UK) and MCU (Abbxa Ltd, abx532522, Cambridge, UK) and SLC8A1 (Abbxa Ltd, abx253795, Cambridge, UK). The protein levels for each sample were normalized to the exosome content of the CD63 exosome marker. The relative value of CD63 for each sample was used to normalize their recovery. The intra-assay and inter-assay coefficients of variation were <10% and <10-12%, respectively. The ELISA microplates were read using the Varioskan LUX 3020 instrument (Thermo Fisher Scientific Oy, Ratastie 2, Vantaa, Finland). Serum samples were analyzed by the same technician, who was blinded to the clinical data.

### Primary outcomes

The primary outcomes were the change of MoCA, PSQI which assessed at baseline, 1, 3, and 6 months after enrollment in RIC group and sham RIC group.

### Secondary outcomes

The secondary outcomes included: (1) the change of HAMD-17, HAMA, the FS-14, ESS which assessed at baseline, 1, 3, and 6 months after enrollment in RIC group and sham RIC group; (2) the changes of MCU, SLC8A1, CFB, and C5b-9 in serum NDEs which quantify at baseline, 1 and 3 months after enrollment in RIC group and sham RIC group; (3) the changes of MoCA and PSQI subdomains which assessed at baseline, 1, 3, and 6 months after enrollment in RIC group and sham RIC group; (4) the difference of MoCA, PSQI, FS-14, ESS, HAMD-17, HAMA and MCU, SLC8A1, CFB, C5b-9 in serum NDEs between insomnia more than 6 months group and insomnia less than or equal to 6 months at baseline.

### Statistical analysis

All data were analyzed using the SPSS software version 26.0. Figures were prepared using GraphPad Prism 9.5.1. *P* = 0.05 was considered statistically significant. Using Kolmogorov-Smirnov test to assess the normality of continuous data. Normal data were expressed as mean ± standard deviation (SD), while the non-normal data were expressed as median and interquartile range (IQR). Univariate analysis was used to evaluate the demographic and clinical characteristics. The differences between groups were assessed via t-test or analysis of variance (ANOVA) for normally distributed variables and the non-parametric Mann—Whitney U test or the Kruskal-Wallis variance test for non-normally distributed variables. Furthermore, the χ2 test was used to compare categorical variables. Age, gender, and educational attainment were included as factors in the repeated measures analysis of the successive changes in cognitive test scores, sleep scale scores, and biomarker levels from serum NDEs for the treatment group and sham group at various time points. The above statistical methods were also used for the analysis of neuropsychological scale and sleep scale subdomains. Moreover, the patients were classified into two groups based on the duration of insomnia at baseline, and an interaction test was performed for subgroup analysis.

## Results

### The baseline characteristics

**Figure 1:**
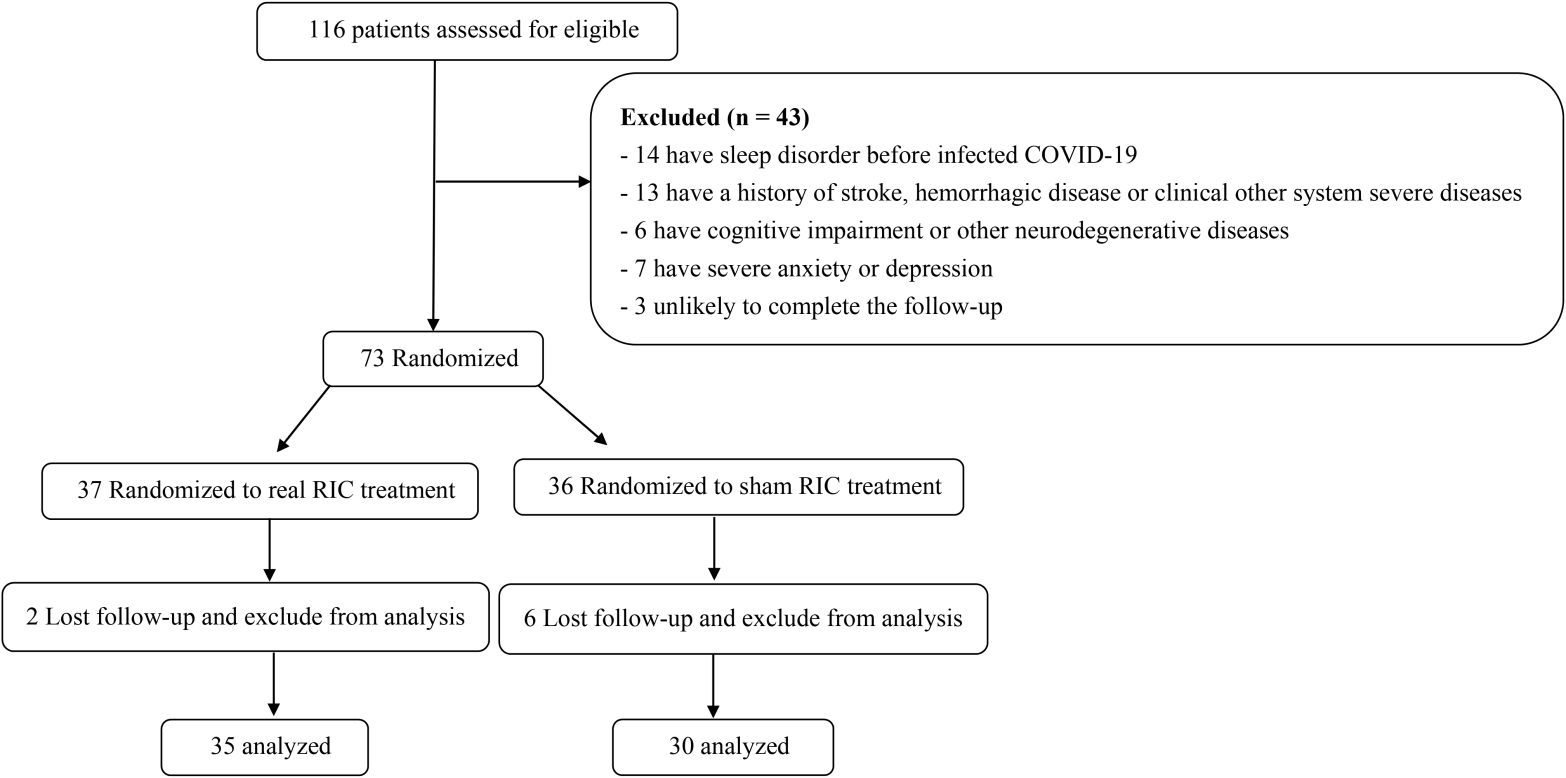
Study flowchart.

A total of 116 patients were enrolled from May 2023 to November 2023. Among them, 14 patients had a history of sleep disorder before COVID-19, 13 patients had stroke and hemorrhagic disease or other severe systemic diseases, 6 patients had cognitive impairment or other neurodegenerative diseases, 7 patients had severe anxiety or depression. Three participants were excluded from the analysis due to incomplete follow-up. Among the 73 randomized individuals, 37 were assigned to the real treatment group and 36 to the sham treatment group. Two patients from the real treatment group and six from the sham group were lost to follow-up and excluded, leaving 65 patients (35 in treatment group and 30 in sham group) for the final analysis. All patients were finished follow-up from June 2023 to May 2024 and there were not important harms or unintended effects in each group (**Figure 1**). Demographic and clinical characteristics are presented in **Table 1**.

**Table 1:** Baseline demographic, laboratory indicators and neuropsychological tests characteristics of all patients before intervention.

	All patients (n = 65)	RIC group (n = 35)	Sham RIC group (n = 30)	*P*
Demographics				
Age, years (mean ± SD)	54.69 ± 10.32	55.09 ± 8.99	54.23 ± 11.83	0.74
Biological Sex, female/male (%)	(86.15/13.85)	(82.86/17.14)	27 (90)	0.49
BMI, kg/m^2^ (mean ± SD)	24.63 ± 3.33	24.44 ± 2.85	24.86 ± 3.86	0.61
Education, years (mean ± SD)	12.22 ± 3.48	12.34 ± 3.12	12.07 ± 3.92	0.75
Hypertension, n (%)	14 (21.54)	6 (17.14)	8 (26.67)	0.35
Diabetes mellitus, n (%)	4 (6.15)	3 (8.57)	1 (3.33)	0.62
Hyperlipidemia, n (%)	20 (30.77)	13 (37.14)	7 (23.33)	0.23
Drinking, n (%)	4 (6.15)	2 (5.71)	2 (6.67)	1.00
Smoking, n (%)	3 (4.62)	2 (5.71)	1 (3.33)	1.00
Laboratory Indicators				
NDEs C5b-9, ng/ml, (mean ± SD)	13.48 ± 5.81	12.87 ± 6.04	14.18 ± 5.54	0.37
NDEs CFB, pg/ml, (mean ± SD)	348.27 ± 43.49	352.52 ± 40.70	343.31 ± 46.74	0.19
NDEs SLC8A1, pg/ml, (mean ± SD)	21.41 ± 18.30	19.96 ± 16.88	23.10 ± 19.98	0.74
NDEs MCU, pg/ml, (mean ± SD)	113.12 ± 82.76	106.99 ± 80.46	120.30 ± 86.18	0.52
Neuropsychological Test				
PSQI scores, (mean ± SD)	9.85 ± 4.78	13.31 ± 2.97	13.31 ± 2.97	0.73
MoCA scores, (mean ± SD)	26.26 ± 2.24	25 ± 2.76	25.13 ± 2.46	0.94
HAMA scores, (mean ± SD)	10.46 ± 3.06	11.17 ± 3.14	10.20 ± 3.41	0.24
HAMD scores, (mean ± SD)	9.09 ± 3.26	9.06 ± 3.13	9.50 ± 3.26	0.58
ESS scores (mean ± SD)	9.74 ± 4.17	9.60 ± 4.60	9.90 ± 3.67	0.78
FS-14 scores, (mean ± SD)	7.68 ± 3.42	9.23 ± 3.26	8.73 ± 3.94	0.71

RIC, remote ischemic conditioning; SD, standard deviation; IQR, interquartile range; BMI, body mass index; NDEs, neuronal-derived exosomes; CFB, complement factor B; MCU, mitochondrial Ca^2+^ uniporterc PSQI, pittsburgh sleep quality index; MoCA, Montreal Cognitive Assessment; HAMD, Hamilton Anxiety Scale; HAMD, Hamilton Depression Scale; ESS, Epworth Sleepiness Scale; FS-14, the 14-item Fatigue Scale.

Univariate analysis was used to evaluate the demographic and clinical characteristics. The differences between groups were assessed via *t*-test or ANOVA for normally distributed variables and the non-parametric Mann—Whitney U test or the Kruskal-Wallis variance test for non-normally distributed variables. The χ2 test was used to compare categorical variables.

### Primary outcomes

**Figure 2:**
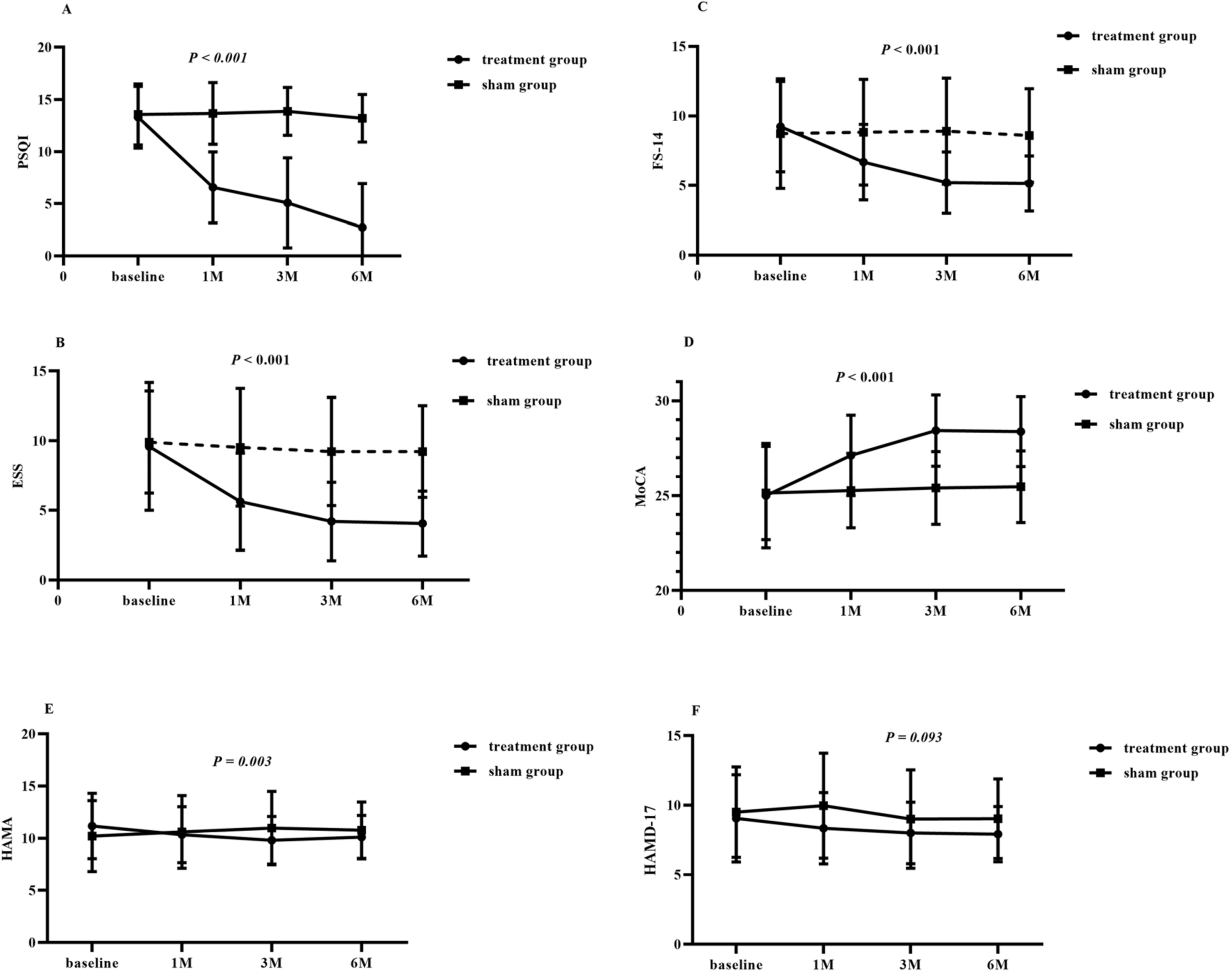
Differences in neuropsychological (**D**, MoCA; **E**, HAMA; **F**, HAMD-17) and sleep scale (**A**, PSQI; **B**, ESS; **C**, FS-14) scores were assessed at each time point (baseline, 1 month, 3 months, and 6 months post-treatment). Abbreviation: 1M, 1 month after treatment; 3M, 3 months after treatment; 6M, the third month after the end of RIC treatment (six months after enrollment); PSQI, Pittsburgh sleep quality index; MoCA, Montreal Cognitive Assessment; ESS, Epworth Sleepiness Scale; FS-14, the 14-item Fatigue Scale; HAMA, Hamilton Anxiety Scale; HAMD-17, Hamilton Depression Scale

PSQI score was compared between the RIC treatment group and the sham group after 1, 3, and 6 months of enrollment based on the baselines. First, the group-by-time interaction showed a significant difference between the two groups (*P* < 0.001). Score of PSQI significantly decreased in the RIC treatment group (30 patients) by 6.74 (*P* < 0.001) after 1 month of treatment. Furthermore, scores of PSQI were significantly decreased in the RIC group by 8.22 (*P* < 0.001) after 3 months of RIC treatment. Similarly, scores of PSQI were significantly decreased in the RIC group by 7.85 (*P* < 0.001) at three months after the end of RIC treatment. Meanwhile, PSQI score was not significantly changed in the sham group after 1, 3, and 6 months of sham RIC treatment (**Figure 2A**, **Table 2**).

**Table 2:** Comparison and repeated measures analysis of neuropsychological assessments and sleep scale scores were conducted for both the treatment and sham groups at different time points.

Scales and biomarkers	Group	Baseline	1 month after treatment	3 months after treatment	6 months after enrollment	*P*					
						[B] vs [1]	[B] vs [3]	[B] vs [6]	Time* group	[3] vs [6]	Time* group
PSQI	Treatment (35)	13.31 ± 2.97	6.57 ± 3.40	5.09 ± 4.32	5.46 ± 3.79	< 0.001*	< 0.001*	< 0.001*	< 0.001*	0.02*	0.39
	Sham (30)	13.57 ± 2.94	13.67 ± 2.98	13.87 ± 2.30	13.20 ± 2.30	0.33	0.16	0.0102			
ESS	Treatment (35)	9.60 ± 4.60	5.63 ± 3.50	4.20 ± 2.82	4.06 ± 2.33	< 0.001*	< 0.001*	< 0.001*	< 0.001*	0.30	0.49
	Sham (30)	9.90 ± 3.67	9.53 ± 4.23	9.23 ± 3.88	9.23 ± 3.31	0.25	0.06	0.057			
FS-14	Treatment (35)	9.23 ± 3.26	6.70 ± 2.72	5.20 ± 2.21	5.14 ± 1.97	< 0.001*	< 0.001*	< 0.001*	< 0.001*	0.48	0.10
	Sham (30)	8.73 ± 3.94	8.83 ± 3.81	8.90 ± 3.82	8.60 ± 3.37	0.38	0.06	0.555			
MoCA	Treatment (35)	25 ± 2.76	27.11 ± 2.13	28.43 ± 1.88	28.37 ± 1.85	< 0.001*	< 0.001*	< 0.001*	< 0.001*	0.16	1.00
	Sham (30)	25.13 ± 2.46	25.27 ± 1.96	25.40 ± 1.92	25.47 ± 1.89	0.54	0.29	0.231			
HAMA	Treatment (35)	11.17 ± 3.14	10.34 ± 2.68	9.80 ± 2.29	10.11 ± 2.10	0.01*	0.003*	0.03*	0.003*	0.04*	0.56
	Sham (30)	10.20 ± 3.41	10.60 ± 3.50	10.97 ± 3.52	10.77 ± 2.71	0.15	0.062	0.09		0.37	
HAMD-17	Treatment (35)	9.06 ± 3.13	8.34 ± 2.57	8.00 ± 2.22	7.91 ± 1.99	0.02*	0.00*	0.02*	0.09	0.32	0.74
	Sham (30)	9.50 ± 3.26	9.97 ± 3.77	9.00 ± 3.54	9.03 ± 2.87	0.19	0.05	0.07		0.85	
C5b-9	Treatment (35)	12.87 ± 6.04	—	10.57 ± 4.89	—	**—**	0.005*	—	0.001*	—	—
	Sham (30)	14.18 ± 5.54	—	15.11 ± 14.75	—	—	0.01*	—		—	
CFB	Treatment (35)	352.52 ± 40.70	—	352.52 ± 40.70	—	—	0.06	—	0.006*	—	—
	Sham (30)	343.31 ± 46.74	—	347.49 ± 43.52	—	—	0.06	—		—	
MCU	Treatment (35)	106.99 ± 80.46	—	189.33 ± 229.98	—	—	< 0.001*	—	< 0.001*	—	—
	Sham (30)	120.30 ± 86.18	—	119.24 ± 95.27	—	—	0.44	—		—	
SLC8A1-3	Treatment (35)	19.96 ± 16.88	—	49.51 ± 32.83	—	—	< 0.001*	—	< 0.001*	—	—
	Sham (30)	23.10 ± 19.98	—	24.18 ± 20.93	—	—	0.25	—		—	

The repeated measures analysis of covariance (ANCOVA) was employed, with age, gender, and education level included as covariates.

*P < 0.05. [B], baseline; [1], 1 month after treatment; [3], 3 months after treatment; [6], the third month after the end of RIC treatment (six months after enrollment); PSQI, Pittsburgh sleep quality index; MoCA, Montreal Cognitive Assessment; ESS, Epworth Sleepiness Scale; FS-14, the 14-item Fatigue Scale; HAMA, Hamilton Anxiety Scale; HAMD-17, Hamilton Depression Scale; NDEs, neuronal-derived exosomes; CFB, complement factor B; MCU, mitochondrial Ca2+ uniporter; SLC8A1, Human Sodium/calcium exchanger 1.

MoCA score was also compared between the RIC treatment group and the sham group after 1, 3, and 6 months of enrollment based on the baselines. First, group-by-time interaction showed significant differences (*P* < 0.001). Compared to baseline, MoCA scores in the RIC group significantly improved by 2.11 (*P* < 0.001) after one month of treatment. After three months of RIC treatment, MoCA scores increased by 3.43 (*P* < 0.001). Additionally, at three months post-treatment, MoCA scores significantly increased by 3.37 (P < 0.001). In contrast, no significant changes were observed in MoCA score in the sham group at 1, 3, and 6 months (**Figure 2D**, **Table 2**).

### Secondary outcomes

ESS, FS-14 scores were compared between the RIC treatment group and the sham group after 1, 3, and 6 months of enrollment based on the baselines. First, the group-by-time interaction showed a significant difference between the two groups (*P* < 0.001). Scores of ESS, and FS-14 significantly decreased in the RIC treatment group (30 patients) by 3.97 (*P* < 0.001), and 2.53 (*P* < 0.001), respectively, after 1 month of treatment. Furthermore, scores of ESS, and FS-14 were significantly decreased in the RIC group by 5.4 ESS (*P* < 0.001), and 4.03 FS-14 (*P* < 0.001), respectively, after 3 months of RIC treatment. Similarly, scores of ESS, and FS-14 were significantly decreased in the RIC group by 5.54 (*P* < 0.001), and 4.09 (*P* < 0.001), respectively, at three months after the end of RIC treatment. Meanwhile, ESS, and FS-14 scores were not significantly changed in the sham group after 1, 3, and 6 months of sham RIC treatment (**Figures 2B, C**, **Table 2**).

HAMA, HAMD-17 scores were also compared between the RIC treatment group and the sham group after 1, 3, and 6 months of enrollment based on the baselines. First, group-by-time interaction showed significant differences (*P* = 0.003 for HAMA). However, HAMD-17 was not significantly different in the group-by-time interaction. HAMA scores decreased by 0.83 (*P* = 0.01) and HAMD-17 scores decreased by 0.72 (*P* = 0.02). After three months of RIC treatment, HAMA scores and HAMD-17 scores decreased by 1.37 (*P* = 0.003) and 1.06 (*P* = 0.005), respectively. Additionally, at three months post-treatment, HAMA and HAMD-17 scores decreased by 1.06 (*P* = 0.03) and 1.15 (*P* = 0.02), respectively. In contrast, no significant changes were observed in HAMA, or HAMD-17 scores in the sham group at 1, 3, and 6 months (**Figures 2E, F**, **Table 2**).

**Figure 3:**
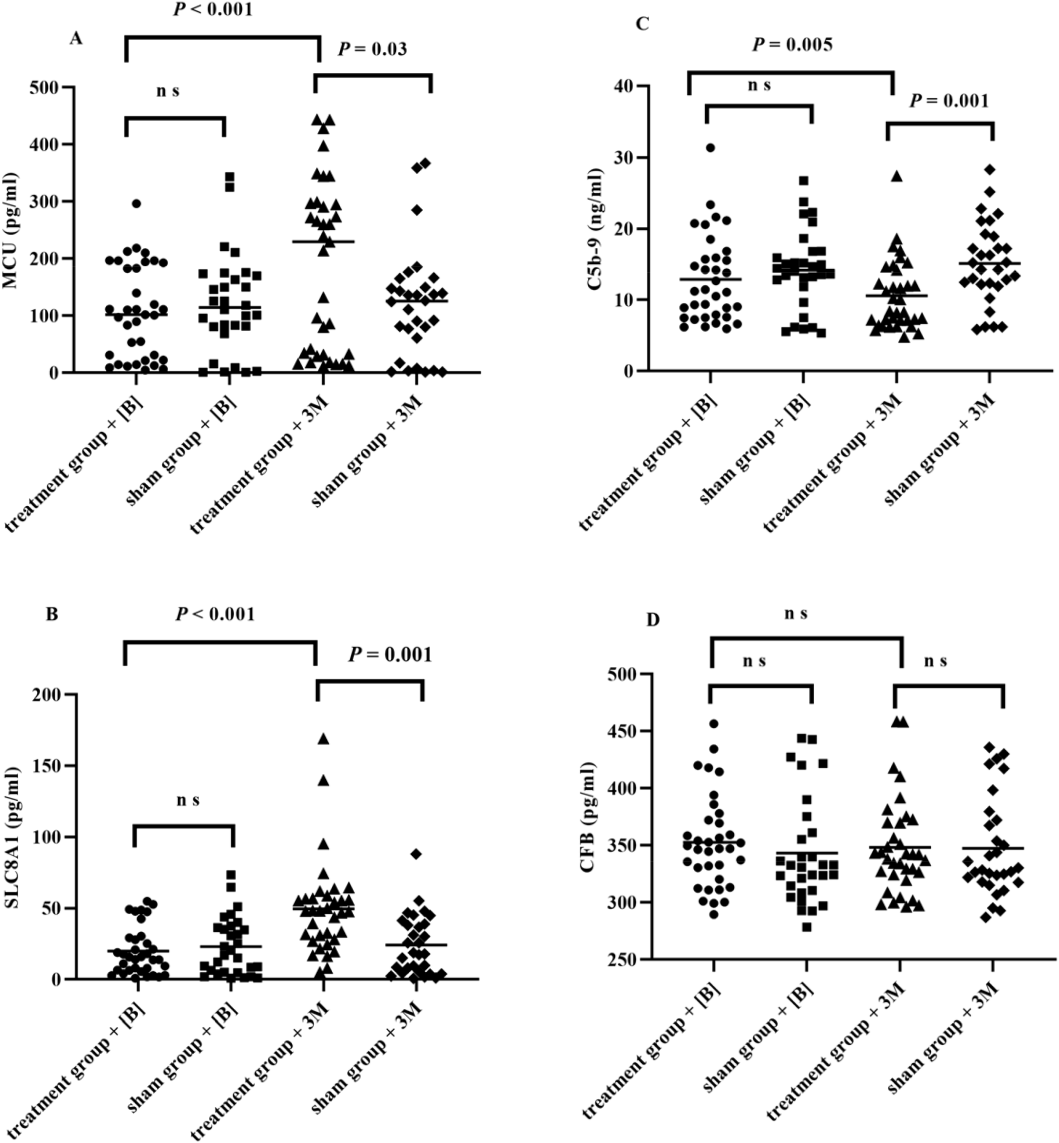
The comparison of MCU **(A)**, SLC8A1 **(B)**, C5b-9 **(C)** and CFB **(D)** in serum NDEs at each time points (baseline, 3 months after treatment). Abbreviation: NDEs, neuronal-derived exosomes;CFB, complement factor B; MCU, mitochondrial Ca2+ uniporter; SLC8A1, Human Sodium/calcium exchanger 1. [B], baseline; 3M, 3 months after treatment.

MCU, SLC8A1, CFB, and C5b-9 in serum NDEs showed significant group-by-time interaction differences (*P* < 0.001, *P* < 0.001, *P* = 0.006, and *P* = 0.001, respectively). Compared with baseline, C5b-9 in serum NDEs significantly decreased in the RIC group by 2.3ng/ml while MCU and SLC8A1 increased by 82.34 pg/ml and 29.55 pg/ml, respectively, after 3 months of RIC treatment (*P* = 0.005, *P* < 0.001, and *P* < 0.001, respectively). Furthermore, C5b-9 in serum NDEs significantly increased in the sham RIC group by 0.93ng/ml after 3 months of sham RIC treatment (*P* = 0.01) compared with baseline. Furthermore, the CFB, MCU, and SLC8A1 levels in serum NDEs were not significantly altered in the sham RIC treatment group after 3 months. Similarly, compared with baseline, CFB levels in serum NDEs were not significantly altered in the RIC treatment group after 3 months (**Table 2**, **Figures 3A–D**).

Differences in PSQI subdomains within the treatment group were evaluated at 1-, 3-, and 6-months post-enrollment, relative to baseline measurements. The results indicated that the group-by-time interaction was statistically significant for sleep quality, sleep latency, sleep duration, sleep efficiency, insomnia, sleep medication use, and daytime dysfunction (*P* < 0.001, *P* < 0.001, *P* < 0.001, *P* < 0.001, *P* = 0.01, *P* < 0.001, *P* < 0.001, respectively). Further analysis revealed that sleep quality, sleep latency, sleep duration, sleep efficiency, somnipathy, sleep medication, and daytime dysfunction were significantly improved after 1 month of RIC treatment (*P* < 0.001, *P* < 0.001, *P* < 0.001, *P* < 0.001, *P* = 0.001, *P* < 0.001, *P* < 0.001, respectively); 3 months of RIC treatment (*P* < 0.001, *P* < 0.001, *P* < 0.001, *P* < 0.001, *P* < 0.001, *P* = 0.001, *P* < 0.001, respectively); and three months after RIC treatment (*P* < 0.001, *P* < 0.001, *P* < 0.001, *P* < 0.001, *P* < 0.001, *P* = 0.001, *P* < 0.001, respectively) compared with the baseline (**Supplementary Table 1**).

Differences in MoCA subdomains within the treatment group were assessed after 1, 3, and 6 months of enrollment based on the baselines. Results showed significant group-by-time interaction differences for visuo-executive, naming, language, and delayed recall (*P* < 0.001, *P* = 0.005, *P* = 0.008, and *P* < 0.001, respectively). Furthermore, the visuo-executive, naming, language, and delayed recall were significantly improved after 1 month of RIC treatment (*P* = 0.005, *P* = 0.006, *P* = 0.009, and *P* < 0.001, respectively). There were significant improvements in the visuo-executive, naming, attention, language, abstraction, delayed recall, and orientation after 3 months of RIC treatment (*P* < 0.001, *P* = 0.002, *P* = 0.03, *P* = 0.005, *P* = 0.02, *P* < 0.001, and *P* = 0.01, respectively). The visuo-executive, naming, attention, language, abstraction, delayed recall, and orientation were also significantly improved at three months after RIC treatment compared with the baseline (*P* < 0.001, *P* = 0.002, *P* = 0.033, *P* = 0.005, *P* = 0.01, *P* < 0.001, and *P* = 0.01, respectively) (**Supplementary Table 2**).

### Exploratory analysis

In the subgroup analysis, there were significant differences in MoCA (*P* < 0.001), PSQI (*P* < 0.001), ESS (*P* < 0.001) scores after 1 month of enrollment and MoCA (*P* < 0.001), PSQI (*P* < 0.001), ESS (*P* < 0.001) scores and SLC8A1 (*P* = 0.01) levels in serum NDEs after 3 months of enrollment. Besides, the interaction effects of MoCA (*P* = 0.002), PSQI (*P* < 0.001), ESS (*P* = 0.001) scores after 1 month and MoCA (*P* < 0.001), PSQI (*P* < 0.001), ESS (*P* < 0.001), HAMA (*P* = 0.04) scores and C5b-9 (*P* = 0.001), SLC8A1 (*P* = 0.001) levels in serum NDEs after 3 months were significant. The detailed results are shown in **Supplementary Table 3** of **Supplementary Material 2**.

### Minimal clinically important differences

According to the distribution method, one-half of the baseline SDs of MoCA, PSQI, ESS, FS-14, HAMA and HAMD-17 were 1.12 and 2.39 and 2.09 and 1.71 and 1.53 and 1.63 for all participants, respectively. According to the anchoring method, the improvement group was defined as an improvement score equal to or greater than the average score of all patients before and after intervention. The anchor-based minimal clinically important differences (MCID) were calculated as the average score change of pre-intervention values and post-intervention value. **Supplementary Table  4** displays the distribution- and anchorbased and MCID estimates for all patients at different time points. The reason why the difference between the distribution method and the anchoring method is too large may be that the patient’s sleep and cognitive impairment improved significantly after RIC treatment.

## Discussion

In this study, RIC improved long-term COVID-19 neuropsychiatric symptoms and inflammation reaction. Furthermore, RIC treatment significantly improved insomnia, cognitive impairment, anxiety, and depression after COVID-19 after three months. RIC treatment markedly decreased the level of C5b-9 in serum NDEs after three months while significantly increasing the levels of MCU and SLC8A1 in serum NDEs. Also, RIC treatment significantly improved insomnia, cognitive impairment, anxiety, and depression symptoms after COVID-19 three months after the end of RIC treatment.

SARS-CoV-2 can enter the CNS through tight junctions by invading host endothelial cells and even across the BBB (33, 34). SARS-CoV-2 can cause injuries to CNS endothelial cells through leukocytes, leading to systematic inflammation and BBB disruption, thus inducing brain structural changes and cognitive impairment (35). SARS-CoV-2 can also aggravate COVID-19-related sleep disturbance by inducing cytokine storms, leading to neuroinflammation and a change in BBB integrity (36). Besides, Marta et al. showed that coronavirus may affect the bioelectrical activity of the brain and perhaps by increasing activity (37). It has been well established that bioelectrical activity of brain was associated with sleep disorder (38), cognitive impairment (39), anxiety and depression symptoms (40). SARS-CoV2 infection can affect the bioelectrical activity of the brain, lower delta band at baseline predict worse cognitive functioning at follow-up (41). Our team has previously demonstrated that the paroxysmal slow wave events were associated with cognitive impairment in patients with obstructive sleep apnea and may related to the inflammatory reaction (42). Previous study also indicated that an imbalance of bioelectrical activity of brain in Anti-LGI1 Encephalitis correlated with the severity of inflammation and cognitive impairment (43). In conclusion, the bioelectrical activity of the brain may involve in insomnia, cognitive impairment, anxiety, and depression in COVID-19 patients, future research is still needed to confirm.

RIC, as a simple and non-invasive and low-cost intervention, triggers the endogenous protective effect of remote organs through short and repeated ischemia-reperfusion treatment of limbs via air sacs. RIC reduces the inflammatory response mediated by intercellular adhesion molecule-1 and endothelin-1, thus improving endothelial cell function and cognitive impairment in patients suffered with acute ischemic stroke (44). RIC can improve cognitive impairment in APP/PS1 rats by protecting BBB integrity and preventing neuroinflammation (45). Previous studies have confirmed that RIC can increase sleep duration in patients with stroke (46) and alleviate cognitive impairment in patients with vascular cognitive impairment. Ma et al. (47) showed that RIC can reduce neuroinflammation, inhibit neuronal apoptosis, improve cognitive impairment, and exert neuroprotective effects by protecting BBB integrity and endothelial function. Herein, results showed that RIC treatment can remarkably improve cognitive impairment, insomnia, anxiety, and depression after COVID-19.

The immune response to SARS-CoV-2 infection in the respiratory system can trigger an autoimmune reaction in the nervous system. This may lead to elevated levels of immune-inflammatory cells, such as cytokines and chemokines, in the cerebrospinal fluid. Moreover, it can activate glial cells in the central nervous system, prompting them to adopt inflammatory phenotypes (2, 48). Under physiological conditions, glial cells can swiftly adapt to the corresponding phenotype in response to immune signals, which facilitates the plasticity of brain circuits and maintaining CNS homeostasis. However, under pathological conditions, dysregulation of glial cells can enhance their phagocytic activity, disrupt neural circuit plasticity and lead to brain injury (36, 49, 50). Exosomes are one of the double-layer membrane-wrapped vesicles exudated by neurons into the extracellular space. Exosomes play a crucial impact in information and material exchange between cells. Exosomes have a small size and cell membrane-like shape and thus can easily pass through the BBB and protect carriers, such as complement proteins and mitochondrial proteins, from degradation, indicating that they can reflect CNS conditions. Notably, complement system and mitochondrial dysfunction are associated with long-term COVID-19 (13, 51). C5b-9 disrupts the cell membrane and causes endothelial cell damage to released substances, such as von Willebrand factor and thrombospondin-1. Meanwhile, the accumulated von Willebrand factor aggregates and activates the complement cascade reaction, thus maintaining local complement activation and aggravating neuroinflammation (52). Previous studies have indicated that C5b-9 is statistically increased in long-term COVID patients compared with patients who recover before 6-month follow-up (13). In our study, C5b-9 in serum NDEs was significantly increased after three months of COVID-19 compared with baseline in the sham RIC treatment group. The MCU protein is the main channel for Ca^2+^ exchange in the mitochondrial matrix (53). SLC8A1, as a mitochondrial sodium-calcium exchanger, protects mitochondrial damage by removing excessive Ca^2+^ from neuronal cells (54). The mitochondria produce a large amount of ROS with increasing Ca^2+^ concentration, leading to neuronal apoptosis (16, 17). The stability of mitochondrial Ca^2+^ concentrations is crucial for both energy production and neuronal survival. SARS-CoV-2, a single-stranded RNA virus, is composed of structural proteins, including the nucleocapsid, spike, envelope, and membrane proteins. Overexpression of the envelope or membrane proteins can selectively disrupt the release of Ca^2+^ from the endoplasmic reticulum, as well as alter the contact sites between the endoplasmic reticulum and mitochondria. This disturbance impairs the homeostasis of the mitochondrial Ca^2+^ signaling pathway, potentially compromising cellular function.^13^ Furthermore, concentration of mitochondrial RNA in the mitochondria of nerve cells significantly increases after infection with SARS-CoV-2 (55, 56). Prior studies have shown that the cytoprotection effect of RIC is due to its effect on mitochondria for the target organ (57, 58) and this beneficial effect may be associated with antioxidation and regulate for Ca^2+^ (59). Mollet et al. also found that the neuroprotection and anti-inflammatory effect of RIC associated with complement system (59). In our study, the level of SLC8A1 in serum NDEs showed a decreasing trend in sham treatment group. Importantly, results suggested that RIC treatment can ameliorate the inflammatory reaction induced by complement proteins after COVID-19. In addition, serum levels of MCU and SLC8A1 in NDEs were significantly elevated following RIC treatment. RIC treatment improved insomnia, cognitive impairment, anxiety, and depression associated with COVID-19, with sustained benefits observed even after the completion of the treatment.

However, our study has several limitations. First, it is a single-center study with a relatively small sample size, highlighting the need for larger, multi-center studies to confirm and validate these findings. Second, the follow-up time after the end of RIC treatment was only 3 months, which is short and cannot clarify the long-term effect after the end of RIC treatment. Third, the blood biomarkers were not evaluated at three months after the end of RIC or sham RIC treatment, and thus the dynamic changes of complement proteins and mitochondria proteins are unknown.

## Conclusion

RIC can reduce complement protein in serum NDEs and improve mitochondrial dysfunction, insomnia, cognitive impairment, anxiety, and depression in insomnia patients after COVID-19. Besides, RIC provides this protective effect even after the end of RIC treatment. Therefore, RIC may be a potential adjuvant therapy for insomnia, cognitive impairment, anxiety, and depression after COVID-19.

## Data Availability

The original contributions presented in the study are publicly available. This data can be found here: https://ngdc.cncb.ac.cn/omix/, accession OMIX014742.
