# Supplementary material for: Remote ischemic conditioning improves neuropsychiatric symptoms in COVID-19 patients: a randomized clinical trial
Source: Front Psychiatry. 2026 Feb 20;16:1674886. doi: 10.3389/fpsyt.2025.1674886 (PMC12963307; doi:10.3389/fpsyt.2025.1674886)
Supplement: Supplementary file 1 [file Supplementaryfile1.docx]

**Clinical study protocol**

**Title:**

Remote ischemic conditioning improves neuropsychiatric symptoms in COVID-19 patients: A Randomized Clinical Trial

**Main investigator:** Yaxuan Wu, MS, Mengfan Li, PhD, Haiying Li, MS, Hui Li, Phd, Tengqun Shen, MS, Yuanyuan Liu, MS, Guangming Wang, MS, Hairong Sun, MS, Zhenguang Li, MS, Jinbiao Zhang, PhD.

**Protocol synopsis**

**Title:** Remote ischemic conditioning improves neuropsychiatric symptoms in COVID-19 patients: A Randomized Clinical Trial

**Study type:** A prospective, single center, double-blinded, Randomized Controlled, Clinical Trial

**Corresponding author:** Jinbiao Zhang, Department of Neurology, Weihai Municipal Hospital, Cheeloo College of Medicine, Shandong University, No. 70. Heping Road, Weihai, Shandong, China.

**Study center:** Weihai Municipal Hospital, Cheeloo College of Medicine, Shandong University

**Ethics:** Authorized by the ethical committee of Weihai Municipal Hospital

**Trial registration:** www.chictr.org.cn (ChiCTR2300071190).

Catalogue

Title: 1

Protocol synopsis 1

1. Study design 4

1.1. Background 4

1.2. Study object 5

1.3. Methods 5

1.3.1. Trial design 5

1.3.2. Participants and sample size 6

1.3.3. Trial flowchart 7

1.3.4. Randomization and masking 8

2. Intervention 8

2.1. Real remote ischemic conditioning 8

2.2. Sham remote ischemic conditioning 9

3. Outcomes measures 9

3.1. Primary outcomes 9

3.2. Secondary outcomes 9

4. Safety monitoring 9

5. Statistical analysis 10

6. Funding 10

7. Data availability 11

8. References 11

1. **Study design**
   1. **Background**

Severe acute respiratory syndrome coronavirus 2 (SARS-CoV2) infection causes acute viral pneumonia, somnipathy [1], cognitive impairment [2], anxiety, depression, and other long-lasting symptoms. The World Health Organization defines long-term coronavirus disease (COVID) as the occurrence of persistent symptoms, such as fatigue and cognitive impairment, after three months of SARS-CoV2 infection, or the appearance of the above clinical manifestations as new symptoms lasting for more than 2 months [3]. Multiple meta-analyses demonstrated that the prevalence of insomnia, cognitive impairment, anxiety, and depression symptoms after SARS-CoV2 infection is estimated to be 12%-47% [1,4–6], 14%-58% [7–9] and 45%-48% [10], respectively. Previous studies have shown that coronavirus disease 2019 (COVID-19) related insomnia significantly increase the anxiety symptom during the COVID-19 pandemic [11]. Li et al. also demonstrating that insomnia was positively associated with anxiety and depression symptom and may independently predicted the occurrence of anxiety and depression symptoms during the first wave of the COVID-19 pandemic in China [12].

Emerging evidence has shown that inflammatory response is involved in long-term COVID, especially the complement system [13] and mitochondria [14]. Meanwhile, C5b-9 disrupts the cell membrane and damages the endothelial cell, thus activating the complement cascade reaction and aggravating neuroinflammation. Furthermore, the mitochondrial Ca^2+^ uniporter (MCU) and human sodium/calcium exchanger 1 (SLC8A1) regulate mitochondria Ca^2+^. Overexpression of structural proteins in SARS-CoV-2 selectively affects the release of Ca^2+^ from the endoplasmic reticulum, thus disrupting the homeostasis of the mitochondrial Ca^2+^ signaling pathway, which increases the level of reactive oxygen species (ROS) and exacerbates neuronal apoptosis [15–17]. Previous studies have shown that C5b-9 expression significantly increases in long-term COVID patients compared with patients who recover before 6-month follow-up [11].

Remote ischemic conditioning (RIC), a promising noninvasive prevention, can protect the endothelium against injury by modulating of systematic inflammation and oxidative stress. It can also improve cognition function in patients with vascular damage and treat insomnia related to Parkinson’s disease [18–19]. Exosomes are small size and cell membrane-like structures with the potential to cross the blood-brain barrier (BBB), transporting information and materials exchange between cells [21–23]. They are secreted by neurons, astrocytes and microglia cells in the central nervous system (CNS). Thus, exosomes can be used as indicator of the actusl condition of CNS. Furthermore, exosomes can carry mitochondrial proteins and complement proteins [24,25].

The current research on COVID-19 mainly focuses on the impact of SARS-CoV2 on the body, with only a few treatment-related studies. This study aimed to explore whether RIC can improve insomnia, cognitive impairment, anxiety, and depression in COVID-19 patients as well as the dynamic changes in C5b-9, complement factor B (CFB), MCU, and SLC8A1 in serum neural-derived exosomes (NDEs) in COVID-19 patients with insomnia before and after RIC treatment.

- 1. **Study object**

To search whether RIC improve sleep, cognition, anxiety, and depression in COVID-19.

- 1. **Methods**
     1. **Trial design**

This prospective, single center, double-blinded, randomized clinical trial was conducted in line with the declaration of the Helsinki guidelines and was approved by the ethical committee of Weihai Municipal Hospital. The trial protocol is shown in Supplement 1. All participants signed informed consent forms. This randomized clinical trial followed the Consolidated Standard of Reporting Trials (CONSORT) reporting guideline. The primary outcomes was whether RIC can improve sleep, cognition, anxiety, and depression symptoms in COVID-19 patients with insomnia? And the secondary outcomes was the effect of RIC for cognitive and sleep subdomain in COVID-19 patients with insomnia.

- - 1. **Participants and sample size**

This randomized clinical trial enrolled COVID-19 patients with insomnia related to SARS-CoV2 infection in Weihai Municipal Hospital from May 2023 to May 2024. COVID-19 was diagnosed based on ORF1ab gene and N gene positive of the new coronavirus nucleic acid test. Assuming a standard deviation of 10 for the neuropsychological score difference between the RIC and sham RIC groups, a superiority test was conducted with 80% power at a 5% significance level (α = 0.05). To account for potential follow-up loss, a total of 73 patients were enrolled, with 36 assigned to each group. The sample size was calculated using the G*Power 3.1 software.

Inclusion criteria:

(1) Patients who developed insomnia three months after COVID-19 and met the diagnosis criteria of the International Classification of Sleep Disorders, Third Edition [26];

(2) Patients aged 30-70 years with no history of neuropsychological assessment;

(3) The participants who signed informed consent forms. Notably, the patients could not use other interventions to treat somnipathy after enrollment.

Exclusion criteria:

(1) Patients with chronic insomnia before COVID-19 according to the diagnostic criteria of the 2017 edition of the Chinese Adult Insomnia Diagnosis and Treatment Guidelines [27];

(2) Patients with dementia before enrollment (the score of the Informant Questionnaire on Cognitive Decline in the Elderly ≥ 3.19) [28];

(3) Patients with bleeding or major surgery, such as aortic or carotid before enrollment;

(4) Patients with fever in the first week before enrollment or hard-to-control severe hypertension (defined as systolic blood pressure > 180 mmHg or diastolic blood pressure > 110 mmHg after drug treatment);

(5) Patients with distal ischemic adaptation contraindications, such as upper limb severe soft tissue injury, fracture or vascular injury, distal peripheral vascular disease, or platelet < 100 × 109 / L;

(6) Patients with severe heart or blood system diseases, thyroid disease, malignant tumor or immune system disease;

(7) Patients whose laboratory test indicators did not qualify (aspartate aminotransferase or alanine aminotransferase higher than the upper limit of normal (thrice); creatinine clearance < 0.6 ml/s; serum creatinine > 265 umol/L (> 3.0 mg/dl);

(8) Patients participating in other clinical studies, or had participated in other clinical researchers within 3 months before enrollment, or showed poor treatment compliance.

(9) Patients taking any of the following drugs that may affect the nervous system: antidepressants, anxiolytics, antipsychotics, glucocorticoids or partial bronchodilators, antiepileptic and may influence cognitive function or sleep drugs (such asbenzodiazepines, non-benzodiazepines, melatonin or melatonin receptor agonists, muscular relaxant, opioid analgesics, barbital, antihistamines, acetylcholinesterase inhibitor, dopamine agonist, monoamine oxidase inhibitor, NMDA receptor antagonists, levodopa, citicoline, piracetam, benzhexol)

- - 1. **Trial flowchart**


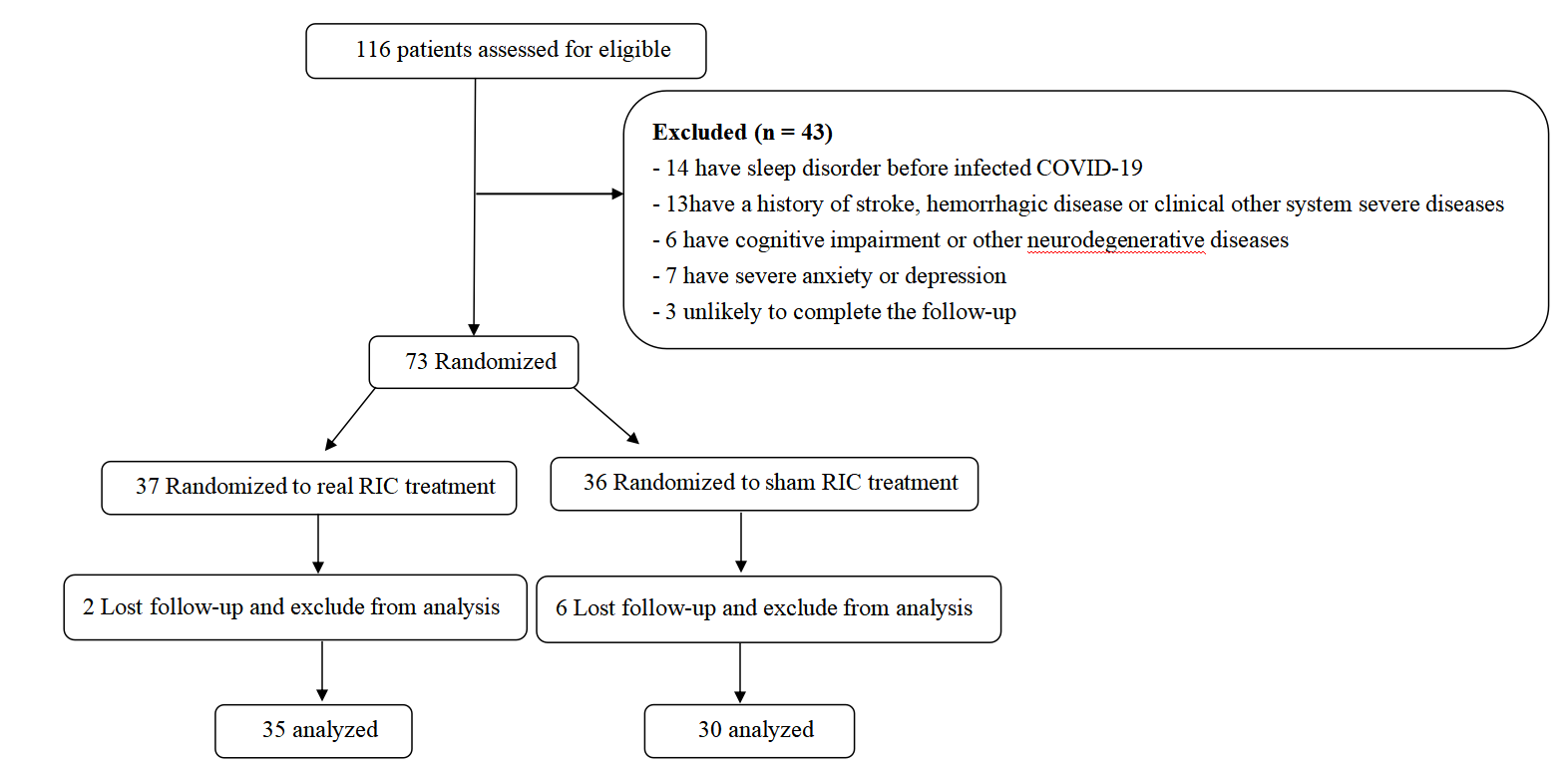


- - 1. **Randomization and blinding**

Patients were randomly assigned to either the RIC treatment group or the sham RIC treatment group using a simple randomization method without stratification. A professional statistician at Weihai Municipal Hospital generated the randomization sequence using SPSS software, with the study sequence concealed in an airtight envelope. Participants and random sequence generator, as well as allocator and evaluators are independent of each other and are not aware of the grouping distribution.

1. **Intervention**
   1. **Real remote ischemic conditioning**

Only RIC equipment from Weihai Guangtai Medical Technology Co. was used in this study. The operation was conducted as follows: bilateral upper arm cuff inflation for 5 minutes, then deflation for 5 minutes. (repeated five times for 50 minutes). The inflation pressure of the upper arm cuff in the RIC treatment group was 200 mmHg, and they continuously received treatment for three months (180 sessions, 2 sessions/day). Similarly, the inflation pressure of the upper arm cuff in the sham-treatment group was 60 mmHg, and they received treatment for 3 months (180 sessions, 2 sessions/day).

- 1. **Sham remote ischemic conditioning**

Only the inflation pressure of the upper arm cuff in the sham-treatment group was 60 mmHg, another operation methods were same to RIC group.

1. **Outcomes measures**
   1. **Primary outcomes**

The primary outcomes were the change of MoCA, PSQI which assessed at baseline, 1, 3, and 6 months after enrollment in RIC group and sham RIC group.

- 1. **Secondary outcomes**

The secondary outcomes included: (1) the change of HAMD-17, HAMA, the FS-14, ESS which assessed at baseline, 1, 3, and 6 months after enrollment in RIC group and sham RIC group; (2) the changes of MCU, SLC8A1, CFB, and C5b-9 in serum NDEs which quantify at baseline, 1 and 3 months after enrollment in RIC group and sham RIC group; (3) the changes of MoCA and PSQI subdomains which assessed at baseline, 1, 3, and 6 months after enrollment in RIC group and sham RIC group; (4) the difference of MoCA, PSQI, FS-14, ESS, HAMD-17, HAMA and MCU, SLC8A1, CFB, C5b-9 in serum NDEs between insomnia more than 6 months group and insomnia less than or equal to 6 months at baseline.

1. **Safety monitoring**

Formulate data security monitoring plan before clinical trial implementation. In the clinical study enrollment and follow-up process, the adverse events of all patients were recorded in detail, timely handled and tracked until the situation was properly resolved or stable, and timely reported to the Ethics Committee

1. **Statistical analysis**

All data were analyzed using the SPSS software version 26.0. Figures were prepared using GraphPad Prism 9.5.1. *P* = 0.05 was considered statistically significant. Using Kolmogorov-Smirnov test to assess the normality of continuous data. Normal data were expressed as mean ± standard deviation (SD), while the non-normal data were expressed as median and interquartile range (IQR). Univariate analysis was used to evaluate the demographic and clinical characteristics. The differences between groups were assessed via *t*-test or analysis of variance (ANOVA) for normally distributed variables and the non-parametric Mann—Whitney U test or the Kruskal-Wallis variance test for non-normally distributed variables. Furthermore, the χ2 test was used to compare categorical variables. Age, gender, and educational attainment were included as factors in the repeated measures analysis of the successive changes in cognitive test scores, sleep scale scores, and biomarker levels from serum NDEs for the treatment group and sham group at various time points. The above statistical methods were also used for the analysis of neuropsychological scale and sleep scale subdomains. Moreover, the patients were classified into two groups based on the duration of insomnia at baseline, and an interaction test was performed for subgroup analysis.

1. **Funding**

This study was supported by grants Qilu Health Leading Talent Cultivation Project：WS-2021-017 and the Shandong Provincial Medical and Health Science and Technology Development Plan (202403071352)

1. **Data sharing statement**

To protect the privacy of all participants, the data in this study cannot be shred publicly. The data will be shared at a reasonable request of the corresponding author.

1. **References**
2. Linh TTD, Ho DKN, Nguyen NN, Hu CJ, Yang CH, Wu D. Global prevalence of post-COVID-19 sleep disturbances in adults at different follow-up time points: A systematic review and meta-analysis. *Sleep Med Rev* 2023;71:101833.
3. Monje M, Iwasaki A. The neurobiology of long COVID. *Neuron* 2022;110:3484–3496.
4. WHO. A clinical case definition of post COVID-19 condition by a Delphi consensus, 6 October 2021. Updated 6 October. Accessed January 31 2025. https://iris.who.int/bitstream/handle/10665/345824/WHO-2019-nCoV-Post-COVID-19-condition-Clinical-case-definition-2021.1-eng.pdf?sequence=1
5. Malik P, Patel K, Pinto C, Jaiswal R, Tirupathi R, Pillai S, et al. Post-acute COVID-19 syndrome (PCS) and health-related quality of life (HRQoL)-A systematic review and meta-analysis. *J Med Virol* 2022;94(1):253–262. doi:10.1002/jmv.27309
6. Alkodaymi MS, Omrani OA, Fawzy NA, Shaar BA, Almamlouk R, Riaz M, et al. Prevalence of post-acute COVID-19 syndrome symptoms at different follow-up periods: A systematic review and meta-analysis. *Clin Microbiol Infect* 2022;28:657–666.
7. Yang T, Yan MZ, Li X, Lau EHY. Sequelae of COVID-19 among previously hospitalized patients up to 1 year after discharge: A systematic review and meta-analysis. *Infection* 2022;50:1067–1109.
8. Badenoch JB, Rengasamy ER, Watson C, Jansen K, Chakraborty S, Sundaram RD, et al. Persistent neuropsychiatric symptoms after COVID-19: A systematic review and meta-analysis. *Brain Commun* 2022;4:fcab297.
9. Giussani G, Westenberg E, Garcia-Azorin D, Bianchi E, Yusof Khan AHK, Allegri RF, et al. Prevalence and trajectories of post-COVID-19 neurological manifestations: A systematic review and meta-analysis. *Neuroepidemiology* 2024;58:120–133.
10. Crivelli L, Palmer K, Calandri I, Guekht A, Beghi E, Carroll W, et al. Changes in cognitive functioning after COVID-19: A systematic review and meta-analysis. *Alzheimers Dement* 2022;18:1047–1066.
11. Deng J, Zhou F, Hou W, Silver Z, Wong CY, Chang O, et al. The prevalence of depression, anxiety, and sleep disturbances in COVID-19 patients: A meta-analysis. *Ann N Y Acad Sci* 2021;1486:90–111.
12. Meaklim H, Burge M, Le F, Bains SK, Saunders W, Ghosh S, et al. Strange themes in pandemic dreams: Insomnia was associated with more negative, anxious and death-related dreams during the COVID-19 pandemic. Journal of sleep research. 2023;32(1):e13655
13. Mu L, Zhou Y, Jamal GC, Wu HE, Wang Y, Wang Y, et al. Insomnia mediates the effect of perceived stress on emotional symptoms during the first wave of the COVID-19 pandemic in China. Journal of affective disorders. 2023;323:770-7
14. Cervia-Hasler C, Brüningk SC, Hoch T, Fan B, Muzio G, Thompson RC, et al. Persistent complement dysregulation with signs of thromboinflammation in active Long Covid. *Science* 2024;383:eadg7942.
15. Davis HE, McCorkell L, Vogel JM, Topol EJ. Long COVID: Major findings, mechanisms and recommendations. *Nat Rev Microbiol* 2023;21:133–146.
16. Poggio E, Vallese F, Hartel AJW, Morgenstern TJ, Kanner SA, Rauh O, et al. Perturbation of the host cell Ca^2+^ homeostasis and ER-mitochondria contact sites by the SARS-CoV-2 structural proteins E and M. *Cell Death Dis* 2023;14:297.
17. Kitao T, Takuma K, Kawasaki T, Inoue Y, Ikehara A, Nashida T, et al. The Na^+^/Ca^2+^ exchanger-mediated Ca^2+^ influx triggers nitric oxide-induced cytotoxicity in cultured astrocytes. *Neurochem Int* 2010;57:58–66.
18. Liu PP, Xie Y, Meng XY, Kang JS. History and progress of hypotheses and clinical trials for Alzheimer's disease. *Signal Transduct Target Ther* 2019;4:29.
19. Orlandi M, Masi S, Bhowruth D, Leira Y, Georgiopoulos G, Yellon D, et al. Remote ischemic preconditioning protects against endothelial dysfunction in a human model of systemic inflammation: A randomized clinical trial. *Arterioscler Thromb Vasc Biol* 2021;41:e417–e426.
20. Ji Q, Wang X, Zhao W, Wills M, Yun HJ, Tong Y, et al. Effects of remote ischemic conditioning on sleep complaints in Parkinson's disease-rationale, design, and protocol for a randomized controlled study. *Front Neurol* 2022;13:932199.
21. Xu R, He Q, Wang Y, Yang Y, Guo ZN. Therapeutic potential of remote ischemic conditioning in vascular cognitive impairment. *Front Cell Neurosci* 2021;15:706759.
22. Vidal M. Exosomes: Revisiting their role as "garbage bags". *Traffic* 2019;20:815–828.
23. Thompson AG, Gray E, Heman-Ackah SM, Mäger I, Talbot K, Andaloussi SE, et al. Extracellular vesicles in neurodegenerative disease - pathogenesis to biomarkers. *Nat Rev Neurol* 2016;12:346–357.
24. Holm MM, Kaiser J, Schwab ME. Extracellular vesicles: Multimodal envoys in neural maintenance and repair. *Trends Neurosci* 2018;41:360–372.
25. Peluso MJ, Deeks SG, Mustapic M, Kapogiannis D, Henrich TJ, Lu S, et al. SARS-CoV-2 and mitochondrial proteins in neural-derived exosomes of COVID-19. *Ann Neurol* 2022;91:772–781.
26. Weng S, Lai QL, Wang J, Zhuang L, Cheng L, Mo Y, et al. The role of exosomes as mediators of neuroinflammation in the pathogenesis and treatment of Alzheimer's disease. *Front Aging Neurosci* 2022;14:899944.
27. Sateia MJ. International classification of sleep disorders-third edition. *Chest* 2014;146:1387–1394.
28. Chinese Society of Neurology, Sleep Disorder Society CSoN. Guideline for the evaluation and treatment of insomnia in Chinese adults (2017). *Chin J Neurol* 2018;51:324–335. (in Chinese)
29. Li F, Jia XF, Jia J. The informant questionnaire on cognitive decline in the elderly individuals in screening mild cognitive impairment with or without functional impairment. *J Geriatr Psychiatry Neurol* 2012;25:227–232.
