# Supplementary material for: Remote ischemic conditioning improves neuropsychiatric symptoms in COVID-19 patients: a randomized clinical trial
Source: Front Psychiatry. 2026 Feb 20;16:1674886. doi: 10.3389/fpsyt.2025.1674886 (PMC12963307; doi:10.3389/fpsyt.2025.1674886)
Supplement: Supplementary file 2 [file Table1.docx]

**eTable 1.** comparisons and repetitive measures analysis of sleep quality scale subdomains in the RIC treatment group at different time point.

| **Subdomains of PSQI scale** | **Changes of scores (mean ± SD)** | | | | ***P*** | | | | |
| --- | --- | --- | --- | --- | --- | --- | --- | --- | --- |
|  | **Baseline** | **1 month after treatment** | **3 month after treatment** | **6 month after enrollment** | **[B] vs. [1]** | **[B] vs. [3]** | **[B] vs. [6]** | **Time* group** | **[3] vs. [6]** |
| Global score | 13.31 ± 2.97 | 6.57 ± 3.40 | 5.09 ± 4.32 | 5.46 ± 3.79 | < 0.001* | <0.001* | <0.001* | <0.001* | 0.02* |
| Sleep quality | 2.14 ± 0.55 | 1.17 ± 0.51 | 1.03 ± 0.79 | 1.09 ± 0.78 | <0.001* | <0.001* | <0.001* | <0.001* | 0.16 |
| Sleep latency | 2.54 ± 0.92 | 1.29 ± 1.10 | 1.06 ± 1.16 | 1.09 ± 0.98 | <0.001* | <0.001* | <0.001* | <0.001* | 0.71 |
| Sleep duration | 2.23 ± 0.84 | 1.17 ±0.99 | 0.94 ± 1.03 | 1.06 ± 0.97 | <0.001* | <0.001* | <0.001* | <0.001* | 0.04* |
| Sleep efficiency | 1.97 ± 1.04 | 0.97 ± 1.04 | 0.40 ± 0.74 | 0.54 ± 0.66 | <0.001* | <0.001* | <0.001* | <0.001* | 0.05 |
| Sleep disturbance | 1.20 ± 0.41 | 0.8 ± 0.47 | 0.54 ± 0.56 | 0.60 ± 0.60 | 0.001* | <0.001* | <0.001* | 0.013* | 0.16 |
| Sleep medication | 0.89 ± 1.16 | 0.06 ± 0.24 | 0.11 ± 0.53 | 0.20 ± 0.58 | <0.001* | 0.001* | 0.001* | <0.001* | 0.08 |
| Daytime dysfunction | 2.34 ± 0.84 | 1.11 ± 0.96 | 1.00 ± 1.06 | 0.89 ± 0.96 | <0.001* | <0.001* | <0.001* | <0.001* | 0.04* |

Used repetitive measures analysis of covariance includes age, gender and education level as covariates for analysis. * *P* < 0.05. Abbreviations: PSQI, pittsburgh sleep quality index; RIC, remote ischemic conditioning; [B], baseline; [1], 1 month after treatment; [3], 3 months after treatment; [6], the third month after the end of RIC treatment (six months after enrollment).

**eTable 2.** comparisons and repetitive measures analysis of cognitive subdomains in the RIC treatment group at different time point.

| **Cognitive subdomains of MoCA scale** | **Changes of scores (mean ± SD)** | | | | ***P*** | | | | |
| --- | --- | --- | --- | --- | --- | --- | --- | --- | --- |
|  | **Baseline** | **1 month after treatment** | **3 month after treatment** | **6 month after enrollment** | **[B] vs. [1]** | **[B] vs. [3]** | **[B] vs. [6]** | **Time* group** | **[3] vs. [6]** |
| Global score | 25 ± 2.76 | 25 ± 2.76 | 28.43 ± 1.88 | 28.37 ± 1.85 | < 0.001* | < 0.001* | < 0.001* | < 0.001* | 0.16 |
| Visuo-executive | 3.54 ± 1.42 | 3.91 ± 1.44 | 4.46 ± 1.27 | 4.46 ± 1.27 | 0.005* | < 0.001* | < 0.001* | < 0.001* | 1.00 |
| Naming | 2.74 ± 0.44 | 2.94 ± 0.24 | 3.00 ± 0.00 | 3.00 ± 0.00 | 0.006* | 0.002* | 0.002* | 0.005* | 1.00 |
| Attention | 5.77 ± 0.49 | 5.77 ± 0.55 | 5.97 ± 0.17 | 5.97 ± 0.17 | 1.00 | 0.03* | 0.03* | 0.34 | 1.00 |
| Language | 2.09 ± 0.72 | 2.40 ± 0.65 | 2.46 ± 0.66 | 2.46 ± 0.66 | 0.009* | 0.005* | 0.005* | 0.008* | 1.00 |
| Abstraction | 1.57 ± 0.66 | 1.77 ± 0.55 | 1.86 ± 0.43 | 1.86 ± 0.43 | 0.05 | 0.02* | 0.01* | 0.51 | 1.00 |
| Delayed recall | 2.91 ± 1.27 | 3.91 ± 1.07 | 4.26 ± 0.98 | 4.20 ± 0.96 | < 0.001* | < 0.001* | < 0.001* | <0.001* | <0.001* |
| Orientation | 5.83 ±0.38 | 5.89 ± 0.32 | 6.00 ± 0.00 | 6.00 ± 0.00 | 0.49 | 0.01* | 0.01* | 0.23 | 1.00 |

Used repetitive measures analysis of covariance includes age, gender and education level as covariates for analysis. * *P* < 0.05. Abbreviations: RIC, remote ischemic conditioning; MoCA, Montreal Cognitive Assessment; [B], baseline; [1], 1 month after treatment; [3], 3 months after treatment; [6], the third month after the end of RIC treatment (six months after enrollment).

**eTable 3.** The subgroup analysis of MoCA, FS-14, ESS, PSQI, HAMA, HAMD-17 scores and MCU, SLC8A1, CFB, C5b-9 levels in serum NDEs between insomnia more than 6 months patients and insomnia less than or equal to 6 months patients. The test for interation was used to analysis.

| **Subgroup** | **RIC (n = 35)** | **Sham RIC (n = 30)** | **Mean difference (95% CI)** | ***P*** | **interaction** |
| --- | --- | --- | --- | --- | --- |
| **MoCA + [B]** | | | | | |
| insomnia more than 6 months | 23 | 23 | 0.261 (-1.282, 1.803) | 0.73 | 0.501 |
| insomnia less than or equal to 6 months | 12 | 7 | -0.774 (-3.262, 1.714) | 0.53 |  |
| **MoCA + 1M** | | | | | |
| insomnia more than 6 months | 23 | 23 | 2.216 (1.063, 3.458) | < 0.001* | 0.002* |
| insomnia less than or equal to 6 months | 12 | 7 | 1.107 (-0.824, 3.039) | 0.25 |  |
| **MoCA + 3M** | | | | | |
| insomnia more than 6 months | 23 | 23 | 3.348 (2.230, 4.466) | < 0.001* | < 0.001* |
| insomnia less than or equal to 6 months | 12 | 7 | 2.464 (0.661, 4.267) | 0.008* |  |
| **PSQI + [B]** | | | | | |
| insomnia more than 6 months | 23 | 23 | -0.043 (-1.804, 1.717) | 0.96 | 0.826 |
| insomnia less than or equal to 6 months | 12 | 7 | -1.012 (-3.852, 1.828) | 0.47 |  |
| **PSQI + 1M** | | | | | |
| insomnia more than 6 months | 23 | 23 | -7.174 (-9.067, -5.281) | < 0.001* | < 0.001* |
| insomnia less than or equal to 6 months | 12 | 7 | -7.381 (-10.343, -4.328) | < 0.001* |  |
| **PSQI + 3M** | | | | | |
| insomnia more than 6 months | 23 | 23 | -8.261 (-10.366, -6.156) | < 0.001* | < 0.001* |
| insomnia less than or equal to 6 months | 12 | 7 | -10.012 (-13.406, -6.617) | < 0.001* |  |
| **ESS + [B]** | | | | | |
| insomnia more than 6 months | 23 | 23 | -0.435 (-2.925, 2.056) | 0.72 | 0.740 |
| insomnia less than or equal to 6 months | 12 | 7 | 0.512 (-3.505, 4.528) | 0.80 |  |
| **ESS + 1M** | | | | | |
| insomnia more than 6 months | 23 | 23 | -4.696 (-6.967, -2.424) | < 0.001* | 0.001* |
| insomnia less than or equal to 6 months | 12 | 7 | -1.750 (-5.413, 1.913) | 0.34 |  |
| **ESS + 3M** | | | | | |
| insomnia more than 6 months | 23 | 23 | -5.522 (-7.508, -3.535) | < 0.001* | < 0.001* |
| insomnia less than or equal to 6 months | 12 | 7 | -3.583 (-6.787, -0.380) | 0.02* |  |
| **HAMA + [B]** | | | | | |
| insomnia more than 6 months | 23 | 23 | 1.348 (-0.523, 3.219) | 0.15 | 0.075 |
| insomnia less than or equal to 6 months | 12 | 7 | 0.810 (-2.208, 3.827) | 0.59 |  |
| **HAMA + 1M** | | | | | |
| insomnia more than 6 months | 23 | 23 | 0.174 (-1.562, 1.909) | 0.84 | 0.05 |
| insomnia less than or equal to 6 months | 12 | 7 | -0.476 (-3.275, 2.323) | 0.73 |  |
| **HAMA + 3M** | | | | | |
| insomnia more than 6 months | 23 | 23 | -0.913 (-2.581, 0.755) | 0.27 | 0.04* |
| insomnia less than or equal to 6 months | 12 | 7 | -1.071 (-1.618, 3.761) | 0.42 |  |
| **HAMD-17 + [B]** | | | | | |
| insomnia more than 6 months | 23 | 23 | -0.391 (-2.301, 1.519) | 0.68 | 0.934 |
| insomnia less than or equal to 6 months | 12 | 7 | -0.452 (-3.533, 2.628) | 0.77 |  |
| **HAMD-17 + 1M** | | | | | |
| insomnia more than 6 months | 23 | 23 | -1.435 (-3.335, 0.465) | 0.13 | 0.230 |
| insomnia less than or equal to 6 months | 12 | 7 | -1.940 (-1.124, 5.005) | 0.21 |  |
| **HAMD-17 + 3M** | | | | | |
| insomnia more than 6 months | 23 | 23 | -0.957 (-2.683, 0.770) | 0.27 | 0.405 |
| insomnia less than or equal to 6 months | 12 | 7 | -0.786 (-1.998, 3.570) | 0.57 |  |
| **C5b-9 + [B]** | | | | | |
| insomnia more than 6 months | 23 | 23 | -1.833 (-5.074, 1.409) | 0.26 | 0.021 |
| insomnia less than or equal to 6 months | 12 | 7 | -1.811 (-7.039, 3.417) | 0.49 |  |
| **C5b-9 + 3M** | | | | | |
| insomnia more than 6 months | 23 | 23 | -3.679 (-6.684, -0.673) | 0.01 | 0.001* |
| insomnia less than or equal to 6 months | 12 | 7 | -7.942 (-12.790, -3.094) | 0.002 |  |
| **CFB + [B]** | | | | | |
| insomnia more than 6 months | 23 | 23 | 13.739 (-11.563, 30.065) | 0.28 | 0.213 |
| insomnia less than or equal to 6 months | 12 | 7 | -10.440 (-51.286, 30.406) | 0.61 |  |
| **CFB + 3M** | | | | | |
| insomnia more than 6 months | 23 | 23 | 3.245 (-21.566, 28.057) | 0.79 | 0.443 |
| insomnia less than or equal to 6 months | 12 | 7 | -12.699 (-52.716, 27.317) | 0.52 |  |
| **MCU + [B]** | | | | | |
| insomnia more than 6 months | 23 | 23 | -22.929 (-72.528, 26.670) | 0.35 | 0.812 |
| insomnia less than or equal to 6 months | 12 | 7 | 8.990 (-71.004, 88.985) | 0.82 |  |
| **MCU +3M** | | | | | |
| insomnia more than 6 months | 23 | 23 | 49.808 (-25.237, 124.853) | 0.18 | 0.099 |
| insomnia less than or equal to 6 months | 12 | 7 | 109.391 (-11.642, 230.425) | 0.07 |  |
| **SLC8A1 + [B]** | | | | | |
| insomnia more than 6 months | 23 | 23 | 0.633 (-10.223, 11.489) | 0.90 | 0.534 |
| insomnia less than or equal to 6 months | 12 | 7 | -11.902 (-29.411, 5.607) | 0.17 |  |
| **SLC8A1 +3M** | | | | | |
| insomnia more than 6 months | 23 | 23 | 20.553 (4.437, 36.668) | 0.01* | 0.001* |
| insomnia less than or equal to 6 months | 12 | 7 | 31.464 (5.472, 57.456) | 0.01* |  |

Abbreviations: *, *P* < 0.05; **, *P* < 0.005; RIC, remote ischemic conditioning; PSQI, Pittsburgh sleep quality index; MoCA, Montreal Cognitive Assessment; ESS, Epworth Sleepiness Scale; FS-14, the 14-item Fatigue Scale; CFB, complement factor B; MCU, mitochondrial Ca^2+^ uniporter; NDEs, neural-derived exosomes; [B], baseline; 1M, one months after RIC or sham RIC treatment; 3M, three months after RIC or sham RIC treatment.

**eTable 4.** Distribution- and anchor-based MCID of RIC and sham RIC group after treatment at different time point.

| Measures | Anchor-based MCID | | | | | Distribution-based MCID |
| --- | --- | --- | --- | --- | --- | --- |
|  | 1M |  | 3M |  | 6M |  |
| MoCA | 2.70 |  | 3.36 |  | 2.58 | 1.12 |
| PSQI | 6.64 |  | 7.34 |  | 6.28 | 2.39 |
| ESS | 3.64 |  | 4.43 |  | 4.67 | 2.09 |
| FS-14 | 3.96 |  | 4.71 |  | 4.27 | 1.71 |
| HAMA | 1.66 |  | 2.29 |  | 2.42 | 1.53 |
| HAMD-17 | 1.75 |  | 1.73 |  | 2.08 | 1.63 |

Abbreviations: RIC, remote ischemic conditioning; PSQI, Pittsburgh sleep quality index; MoCA, Montreal Cognitive Assessment; ESS, Epworth Sleepiness Scale; FS-14, the 14-item Fatigue Scale; HAMD, Hamilton Anxiety Scale; HAMD, Hamilton Depression Scale; 1M, one months after intervention; 3M, three months after intervention; 6M, the third month after the end of intervention (six months after enrollment); MCID, minimal clinically important differences.
